# Supplementary material for: Disease-associated genotypes of the commensal skin bacterium Staphylococcus epidermidis
Source: Nat Commun. 2018 Nov 28;9:5034. doi: 10.1038/s41467-018-07368-7 (PMC6261936; doi:10.1038/s41467-018-07368-7)
Supplement: Supplementary file 1 — Supplementary Information [file 41467_2018_7368_MOESM1_ESM.pdf]

Supplementary information

Disease-associated genotypes of the commensal skin bacterium *Staphylococcus epidermidis* - Meric et al.

Supplementary methods

Pangenome-wide association study (pGWAS) and functional filtering to identify candidate adaptive genetic traits in *S. epidermidis*.

Phenotypic groups of isolates defined based upon isolate source: asymptomatic carriage vs. infection isolates. An accurate phylogeny of the whole genomic dataset was reconstructed using ClonalFrameML (Figure 1) and a combination of 2×76 pairs of isolates from both groups clustering at the tip of tree branches were selected for conducting two technical replicates of the genome-wide association (Figure S6). Brief steps are described below, and a full detailed procedure for the method, including all scripts, can be found on https://github.com/sheppardlab/pGWAS.

(a) Automatic annotation of genomes and creation of a pan-genome list

This step, based on previously published method (1) allows the creation of a reference-free list of all unique genes and their various alleles present in the genome dataset. The assembled genome contig. files were automatically assembled with RAST (2, 3) and the ***pangenome.pl*** Perl script (https://github.com/sheppardlab/pGWAS) was used to run BLAST to compare every gene (CDS) to every other entry of this gene list. CDS sharing more than 70% sequence identity over > 10% of the sequence length were considered the same gene. Genec were considered different when the local alignment identity fell below this threshold (1).

(b) Brief principle, input files and pGWAS execution

Genomes in pGWAS were fragmented into consecutive overlapping 30-bp kmers, of which the prevalence was statistically compared in isolates from one phenotypic group of interest to another. Based on a previously published approach (4, 5), this method is alignment-free and allows the detection of genomic variants, whether caused by point mutation, homologous recombination, or lateral gene transfer, and controls for the effect of population structure and the possible clonal inheritance of genetic variants by simulating random word evolution and comparing the simulation with observed distributions on the tree (4, 5). Our method has been improved by performing the association and mapping the whole dataset pangenome, rather than a single reference genome, making our approach also entirely reference-free. Input files required for our scripts (available on https://github.com/sheppardlab/pGWAS) were the assembled genomes (contigs), a guiding phylogenetic tree in Newick format, a reference pangenome file (in FASTA and BED formats) and an isolate group text file to correspond isolate identifiers with phenotypic groups to compare. The pGWAS was executed using the ***assomap_given_phylo.py*** script for each technical replicate dataset.

(c) Mapping of associated kmers to a pangenome annotation

The output files from the previous step were processed (for each replicate) using the ***summarize_assoc_words.pl*** script, producing 9 output files summarising the hits for the corresponding pGWAS run. Files with the suffix ".overlapGenes" contained all kmers associated with the groups of interest, mapped to corresponding genes from the pangenome, and including a score of association for each kmer. The overlap from results of 2 independent technical runs were extracted for further analysis and interpretation.

(d) Correlation of GWAS hits with secondary *in vitro* or *in vivo* phenotype scores

As genotype-phenotype maps for complex phenotypes typically involve many genes in functional networks, the GWAS analysis of phenotypes linked to broad ecological data. Virulence or complex physiological processes can produce low-significance hits, without distinct significantly associated genotypes. Here, we used a novel approach to correlate secondary phenotypic scores performed on the same strains used for GWAS with associated genotypic information from step (c) above. Specifically, we tested *in vitro* biofilm formation, methicillin resistance, cell toxicity, and *in vivo* production of interleukin-8 (IL8) by HaCaT keratinocytes and human blood serum, and correlated (Fisher's exact test) higher and lower percentiles of quantitative phenotype scores with the prevalence of associated kmers obtained from step (c). For this, input files included the associated kmer sequences in multi-FASTA format, the assembled genome files (contigs) of the isolates used in the secondary phenotype experiments, and a secondary phenotype score text file containing the results of phenotypic experiments ranked and categorised in 3 percentiles (low/medium/high). Three consecutive scripts were run to process the data and calculate Fisher correlation scores (instructions available on: https://github.com/sheppardlab/pGWAS): the ***1_split_words_per_gene.pl*** script was run on the input files described above, the ***2_multi_count_words_per_word.pl*** script on the output of the first script, and finally the correlation R script ***3_correlation_script_V2.R*** to perform the Fisher correlation between the prevalence of every associated kmer with corresponding secondary phenotype scores. The resulting list of functionally-filtered associated genetic elements contained was ranked by Fisher's exact test *p* values and Manhattan plots were visualised to identify the top hits for subsequent interpretation and analysis.

Biofilm formation assay.

A previously published method for quantifying *in vitro* biofilm formation was adapted (6). Briefly, we used crystal violet to stain bacteria attached to the polystyrene surface of a 96-well microtitre plate, in 3 biological replicates for each bacterial strain. For each strain, 100 µl of tryptone soy broth (TSB) was inoculated with 30 µl aliquots of overnight cultures (OD_{595} between 1.0 and 1.5) in a 96-well plate. Plates were incubated at 37°C for 24 hr. Culture medium was removed and the wells washed with PBS. Plates were air-dried and then stained with 130 µl of 0.1% (w/v) crystal violet for 30 min. Excess stain was removed and the wells washed with PBS, adhered bacteria were air-dried and 130 µl of ethanol:acetone (70:30 w:w) was added and incubated for 10 min at room temperature. OD_{595} were determined for all wells using a BMG Omega spectrophotometer (BMG Labtech, Germany) after the bound dye was dissolved using ethanol:acetone. The final biofilm formation value was calculated by subtracting the median absorbance of the three parallels of the control from the median absorbance of blanks (7).

Vesicle formulation and toxicity assay.

This assay is based on earlier studies using phospholipid vesicles encapsulating self-quenched fluorescent proteins to report the presence and quantify the effect of bacterial cytolytic toxins *in vitro* (8-10). Briefly, vesicle suspensions were prepared by mixing lipid and fatty acid components in chloroform: 25 mol % of 10,12-Tricosadiynoic acid, 53 mol % of 1,2-dipalmitoyl-sn-glycero-3-phosphocholine, 2 mol % of 1,2-dipalmitoyl-sn-glycero-3-phosphoethanolamine and 20 mol % of cholesterol. All chemicals were purchased from Sigma-Aldrich and lipids from Avanti Polar lipids. To incorporate the fluorescent protein into vesicles, the dried lipid was rehydrated using 10 mL of 50 mM 5(6)-carboxyfluorescein (CF) in HEPES buffer solution, vortexed and heated at 75°C for 10 minutes. The solution was then homogenised by three freeze/thaw cycles in liquid N_2 until a turbid solution was obtained, indicative of vesicle formation. Vesicles were extruded using a Liposofast vesicle extruder as previously reported (9), until a translucent solution was obtained, and uncapsulated CF was removed by purification through illustra Nap-25 columns (GE Healthcare), also used for washing steps (9). After treatment, vesicles were finally cross-linked using a CL1000 Ultraviolet crosslinker.

The cytotoxicity of various *S. epidermidis* strains was assessed using a supernatant assay variant of our vesicle toxicity assay (9). Briefly, bacteria were grown for 18h, and the culture

supernatant was harvested by centrifugation at 14,000 rpm for 10 min and filter sterilized through a 0.22 µm filter. Fifty microliters of vesicle solution prepared as above were incubated with 50 µl of bacterial supernatant and fluorescence intensity was measured for 30 min at excitation and emission wavelengths of 485–520 nm respectively on a FLUOROstar fluorimeter (BMG Labtech). Normalised fluorescence values were obtained using the equation $\frac{F_t - F_0}{F_m / F_0}$ where F_t is the average fluorescence value at a specific time point, F_0 is the minimum and F_m is the maximum fluorescence value in the whole run.

Immunological quantification of IL-8 levels in keratinocytes and blood serum in response to *S. epidermidis* infection.

Production of immune response markers by a keratinocyte cell line from human skin epithelial, and by human blood serum was measured by ELISA after challenge by 80 strains of *S. epidermidis* representing the genomic diversity of the species (11), 43 of which were included in the GWAS runs. For both assays, single overnight colonies were used to inoculate 5 ml of TSB, which were incubated for 16 hours at 37°C with agitation (200 rpm). Bacterial cells were washed in PBS and the solution was diluted to a final concentration of ~10^7 CFU/ml (OD=0.1) in PBS, prior to infection of cells.

(a) HaCaT keratinocytes infection.

HaCaT keratinocytes (12) were cultured in Dulbecco's Modified Eagle's Medium (DMEM, Invitrogen) containing 10% FBS, 1% penicillin/streptomycin and 1% L-Glutamine. HaCaT cells were seeded at 3×10^6 cells/T175 flask and incubated overnight, at 37°C in a 5% CO_2 atmosphere. Cells were washed and resuspended in antibiotic-free Dulbecco/Vogt Modified Eagle’s Medium (DMEM) containing 1% FBS and 1% L-Glutamine, and were then incubated for 72h at 37°C. HaCaT cells culture media was washed and replaced with new batch of antibiotic-free media containing bacteria. In each well of 24-well plates, a 1:2 cell/bacteria ratio was made in 500 µl of culture, which contained 5×10^4 cells/well (in 450 µl) and 2.5×10^4 bacteria (in 50 µl). Incubation lasted 4 hours at 37°C. The supernatant was centrifuged at 13,000 rpm for 5 minutes and frozen at -20°C before quantification of immune markers by ELISA.

(b) Human blood serum infection.

Human blood serum from healthy volunteers was isolated using the vacuette blood collection system (5 ml to 9 ml) on the day of the experiment. Volunteers gave their consent, as assessed by the local Human Tissue Act committee (Wales REC 6) at the Swansea University Medical School (ref: #13/WA/0190). Fifty microliters of *S. epidermidis* overnight cultures were added

to 1 ml of whole blood, at a final concentration of ~5×10^5 CFU/ml. Infected blood was incubated on a rotation wheel at 10 rpm at 37˚C for 4 hours. Samples were then centrifuged at 13,000 rpm for 5 min before the supernatant was decanted and frozen at -20°C before quantification of immune markers by ELISA.

(c) Quantification of immune markers by ELISA.

Duoset ELISA (R and D Systems, Abingdon) for IL-8 was carried out according to the manufacturers' instructions. The capture antibody (mouse anti-human IL-8) was diluted to a working concentration of 4µg/mL in PBS, without carrier protein. A half area 96-well plate was coated with 50µL per well of the diluted capture antibody and incubated at room temperature overnight. Each well was aspirated and washed three times with 150 µL of wash buffer (0.05% Tween 20 in PBS). The well plates where blocked by adding 150µL of block buffer (1% BSA in PBS) to each well for an hour. Each well was aspirated and washed three times with 150 µL of Wash Buffer. 50µL of samples and standards (seven point standard curve with a high standard of 2000pg/mL) were added to the plate and incubated in room temperature for an hour. Infected blood sera were diluted 1/10 prior to ELISA procedure. Aspiration/washing step was repeated. 50µL of the detection antibody (biotinylated goat anti-human IL-8), diluted to a working concentration of 20ng/mL, were added to each well and incubated in room temperature for an hour. Aspiration/washing step was repeated. 50µL of the working dilution of Streptavidin-HPR were added to each well and incubated in room temperature for 20 minutes. Aspiration/washing step was repeated.50µL of substrate solution (1:1 mixture of H_2O_2 and tetramethylbenzidine) was added to each well incubated in room temperature for 20 minutes avoiding placing the plate in direct light. 50 µL of stop solution (2 N H_2SO_4) was added to each well. The optical density of each well was determined immediately using a microplate reader set at 450nm. The wavelength correction was set to 570nm. A standard curve was calculated using a computer generated 4-PL curve fit. Human IL-8 concentrations of the samples were determined based on the standard curve.

Supplementary Notes

We present here a novel approach to functionally filter bacterial GWAS hits from a complex trait (i.e. *S. epidermidis* pathogenicity) using scores of relevant secondary phenotypes. In the scope of this study, we tested for phenotypes that have been described as relevant to staphylococcal virulence, such as biofilm formation (13, 14), methicillin resistance (15), cell toxicity (9, 16) and post-infection interleukin-8 (IL-8) levels in skin epithelial cells and blood serum (17-19). We filtered associated genetic markers by identifying elements overrepresented in isolates that where *in vitro* assayed in four different infection related phenotypes. Many genes that had been previously associated with the corresponding phenotype were identified in the gene lists (Supplementary Data 1), which strongly highlighted that our method of functional filtering was robust for identifying functions associated with important phenotypes in bacteria. A detailed description of genes containing elements associated with pathogenicity and correlated with high virulence-related secondary phenotype scores is given below.

(a) Genes correlated with high levels of IL-8 production in human blood after infection

Within the IL-8 correlated genes (Supplementary Data 1) we found genes encoding proteins already associated with infection and IL-8 modulation in various studies. Among them glucose 6-phosphate dehydrogenase (*SE0704*, *G6PD*) has the third highest score in our list, a proven modulator of IL-8 secretion during inflammation (20, 21). In addition, the list contained fibronectin and fibrinogen binding proteins that have been found important in *S. aureus* to stimulate inflammation in the early steps of an infection (22-24).

(b) Genes correlated with high levels of biofilm formation in vitro

Among the genes correlated with biofilm formation (Supplementary Data 1) was a well-studied biofilm mediator polysaccharide intercellular adhesin (PIA) *icaA* encoded by the *ica* operon (25). An accumulation-associated protein (*aap*) that mediates biofilm formation in the absence of PIA (26) was found to be correlated below the $-\log_{10}$(Fisher *p*)=3.5 threshold (data not shown), but still correlated with lower power. Additionally, *pckA* encoding a phosphoenolpyruvate carboxykinase has been found to aid the synthesis of capsular and intercellular polysaccharides in environments lacking glucose (27, 28). Correlated additional genes also comprised genes that have been either directly related with biofilm formation or with bacterial metabolism in a biofilm environment. Treatments for biofilm infections using inhibitors of histidine metabolism (*yycG*, *hisA*), proteins essential to *S. epidermidis* growth under natural conditions, have been already proposed (29). Lipase encoding genes (*SE0281*, *geh-1*) have been found to be among the up-regulated genes involved in biofilm formation (30).

(c) Genes correlated with high levels of cell toxicity from an in vitro vesicle assay

Nine genes from the arginine catabolic mobile element (ACME) island harboured 30-mers that were associated with pathogenicity and correlated with cell toxicity scores *in vitro*, which represented half of all genes correlated with this phenotype (Supplementary Data 1). The presence of the ACME genomic island has been linked to increased capacity to colonise the human skin and mucosal surfaces in *S. aureus* (31, 32), in which it has been hypothesised to have been horizontally transferred from *S. epidermidis* (32). Moreover, the mobilisation of the ACME is believed to be regulated by the cassette chromosome recombinases (*ccrAB*) from the SCC*mec* island (32-34), which makes these two genomic islands ecologically linked in *Staphylococcus* species (33). The master virulence regulator *agrC* gene was also correlated with cell toxicity scores, and has been shown to be responsible for the synthesis of proteins including hemolysins and toxic shock syndrome toxin. Additionally, it has also been linked to the particular vesicle assay we used in a previous study (31, 35, 36). A universal stress protein (*uspA*) in our list was found to be induced in response to stress conditions during an infection such as starvation, temperature shock and presence of agents that arrest cell growth (37).

(d) Genes correlated with methicillin resistance in vitro

The methicillin resistance phenotype was associated with the highest correlation p-values in our dataset (Figure 3F, Supplementary Data 1). Many of the corresponding genes were part of the well-known SCC*mec* resistance cassette, a common mobile element in *Staphylococcus sp.* but also of the ACME composite island (38). These genes included *mecA* which encodes a penicillin binding protein conferring methicillin resistance, *mecR1* which controls the expression levels of *mecA*, and *ccrB*, encoding a cassette chromosome recombinase (39). A DNA gyrase gene (*gyrB*) associated with fluoroquinolone resistance was also correlated.

Overall, we found high statistical correlation between many well characterised infection associated genes and four *in vitro* assayed virulence related phenotypes, a strong indication that our approach can detect genes with roles in virulence. We detected many previously published virulence factors that promote infection directly, as well as, genes that assist in the adhesion, colonisation, metabolism and antibiotic resistance of the bacteria during infection. Those traits are presumed important for the survival of the bacteria during infection. Many of those genes were already associated with the corresponding phenotype in the literature but our findings also included hypothetical proteins and annotated genes that have not previously been linked with virulence and infection, possibly providing with novel approaches for diagnostics, treatment or even putative novel drug targets.

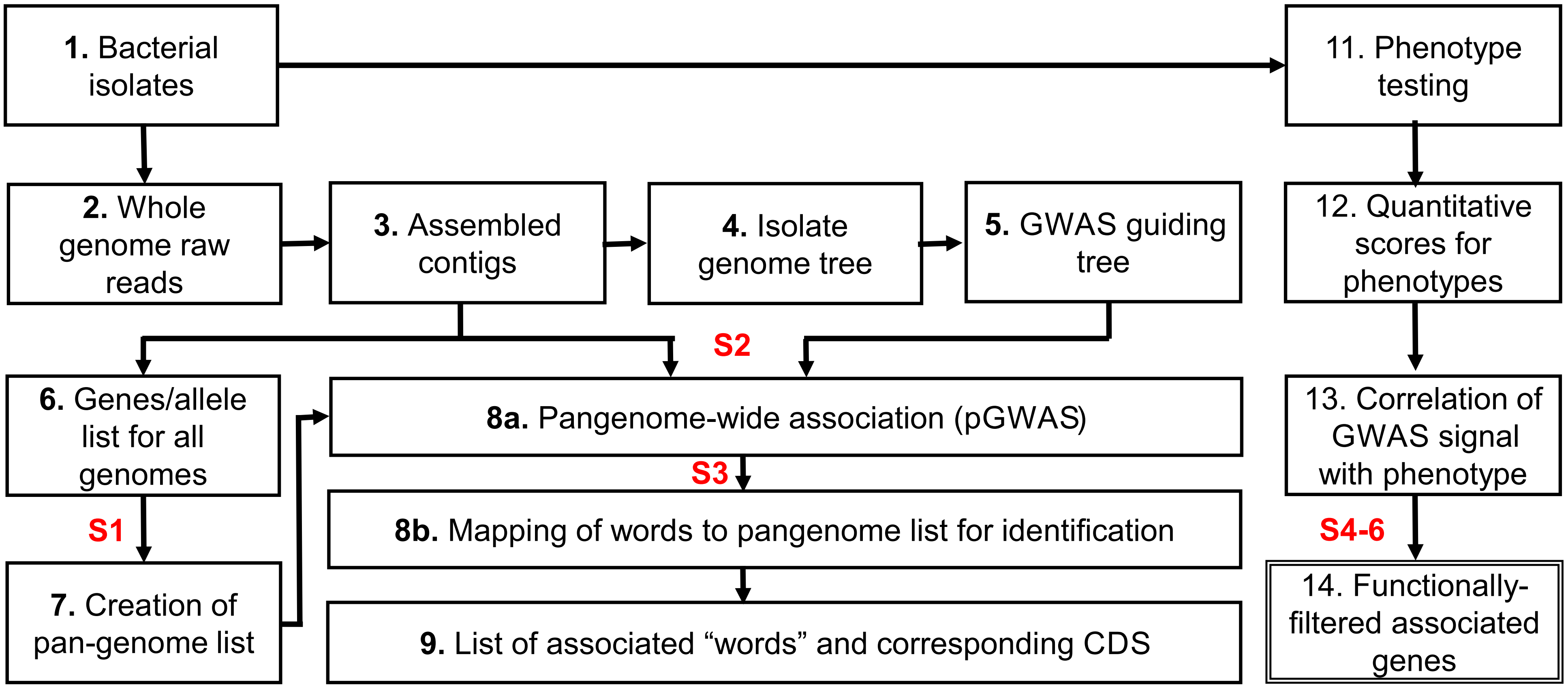

Supplementary Figure 1. Pangenome-wide association study and functional filtering pipeline to identify candidate adaptive genetic traits in *S. epidermidis*. Approach used in this study, including detailed steps and scripts used (red). All scripts used in this study can be found on: https://github.com/sheppardlab/pGWAS.

Scripts used in this pipeline
S1. pangenome.pl
S2. assomap_given_phylo.py
S3. summarize_assoc_words.pl
S4. split_words_per_gene.pl
S5. multi_count_words_per_word.pl
S6. correlation_script_V2.R

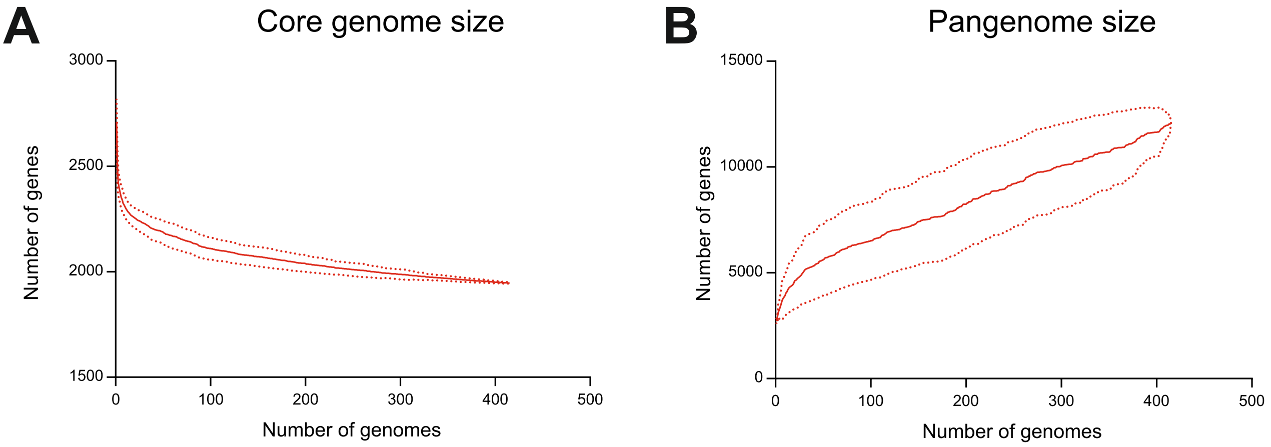

Supplementary Figure 2. Rarefaction and accumulation curve estimates of *S. epidermidis* core and pan-genomes. Number of shared genes ("core genome", A), and the total number of genes (B, "pangenome") were determined as genome sampling increased. Comparisons were made based on matrices of gene presence/absence, derived from the reference pangenome list, for 415 *S. epidermidis* genomes used in this study. Randomized genome sampling was carried out 100 times to obtain the average number of genes for each sample comparison number (plain lines) and standard deviations (dotted lines).

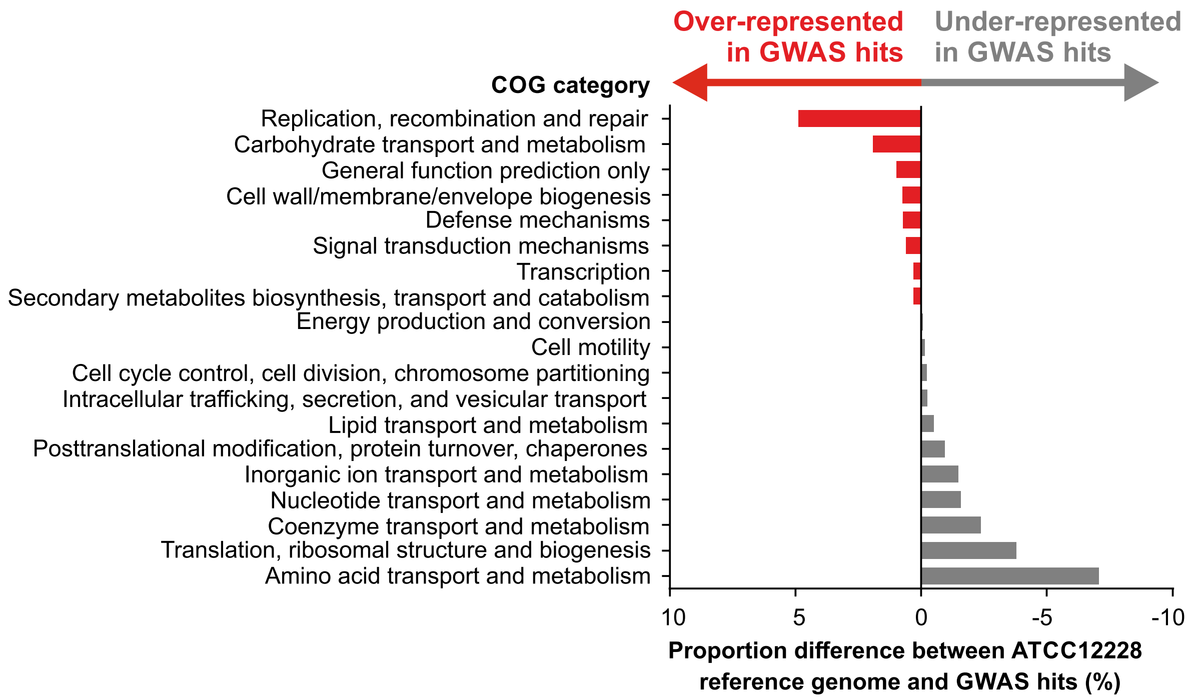

Supplementary Figure 3. Functional categories of genes containing pathogenicity-associated kmers. Attribution of 2,416 genes from the reference *S. epidermidis* ATCC12228 genome and 636 genes containing pathogenicity-associated kmers identified by GWAS to COG families was performed. The proportion of genes allocated to each COG category (%) was compared for the two groups and +/- values indicate deviation from parity.

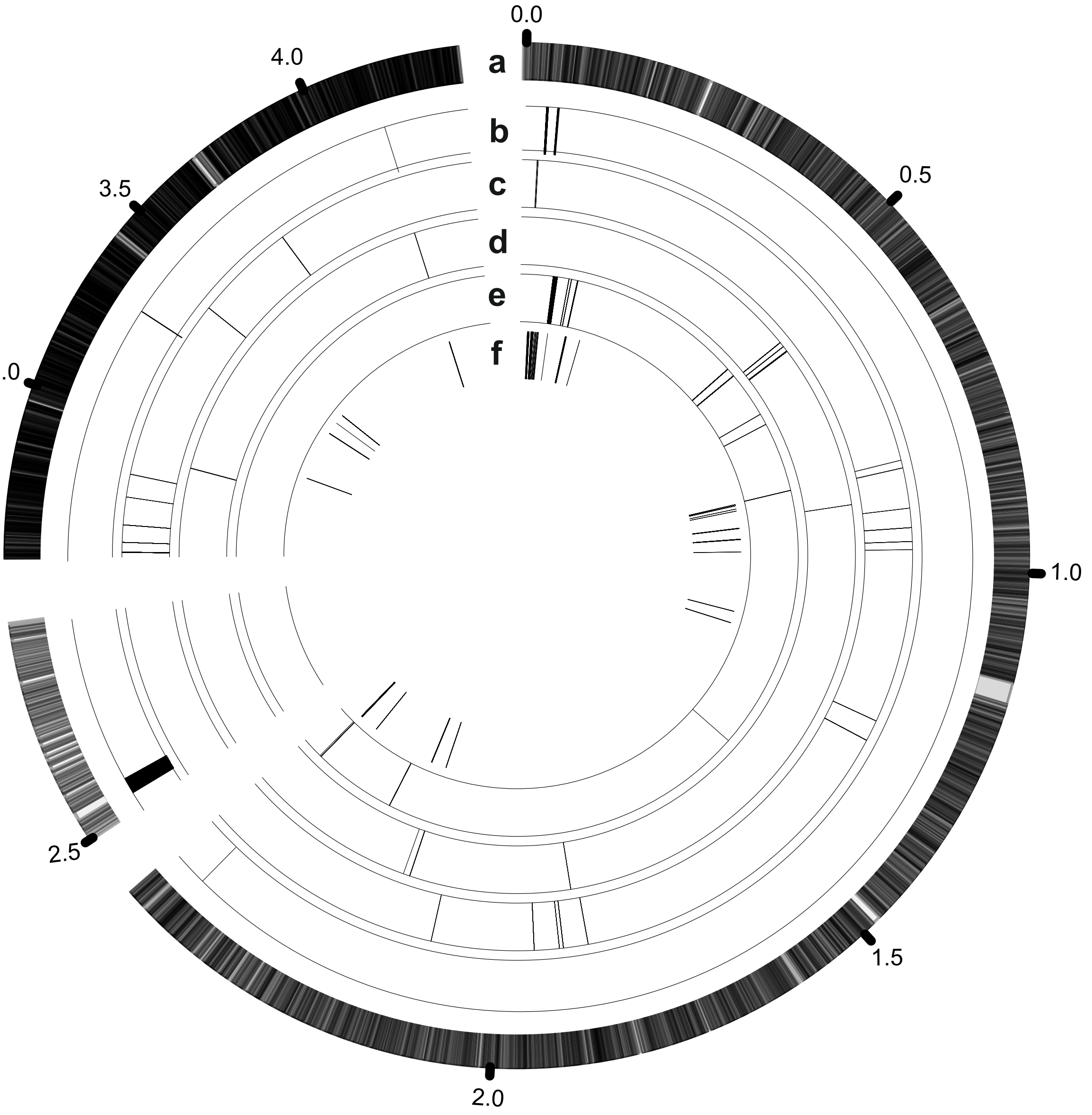

Supplementary Figure 4. Pangenomic positions of associated k-mers of interest after correlation with secondary *in vitro* phenotypes scores. Concentric circles show, in order from outer to inner: (a) pangenome annotation as in Figure 1C; (b) k-mers correlated with methicillin resistance [-log(Fisher's *p*)>10], n = 8424; (c) k-mers correlated with biofilm formation [-log(Fisher's *p*)>3.5, n=2169; (d) k-mers correlated with IL-8 production in human blood serum after infection [-log(Fisher's *p*)>3.5], n=489; (e) k-mers correlated with cytotoxicity using a vesicle assay [-log(Fisher's *p*)>3.5], n=9465; (f) k-mers correlated in more than one phenotype [no *p* threshold], n=3061.

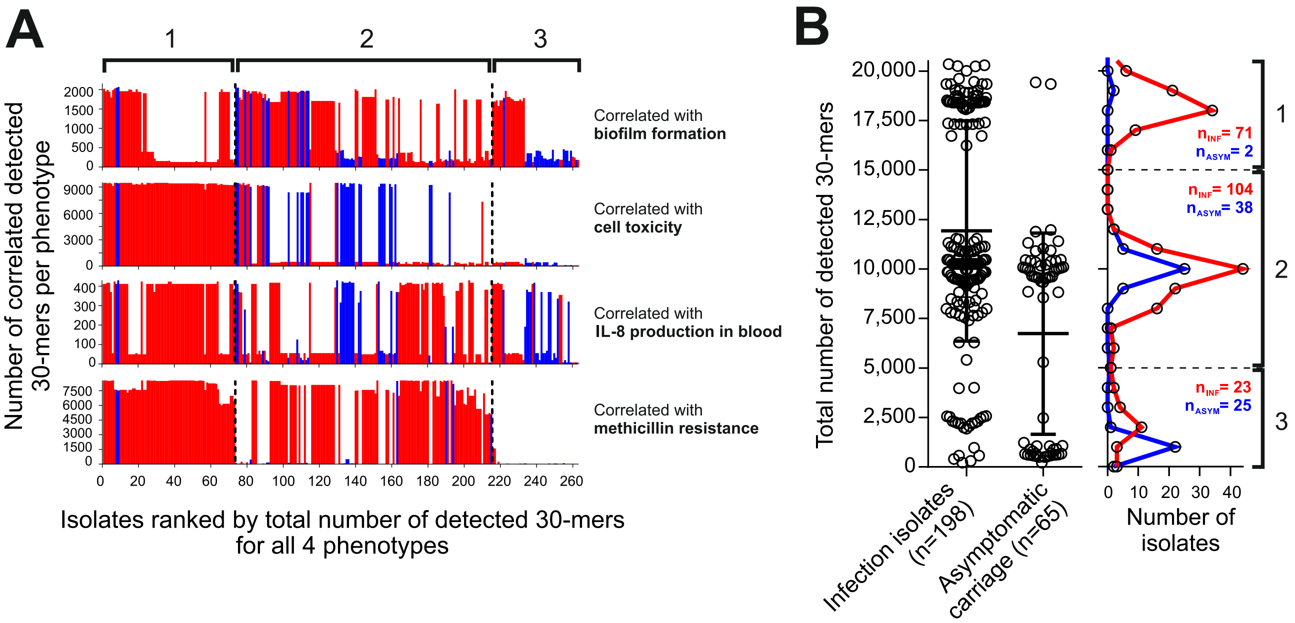

Supplementary Figure 5. Prevalence of disease-causing k-mers in a validation dataset of 263 *S. epidermidis* isolates not used for GWAS or phenotyping. (A) BLAST was used to determine the prevalence of 2168 associated k-mers correlated with biofilm formation, 9477 with cell toxicity, 488 with IL-8 production in blood and 8520 with methicillin resistance, in 198 infection (red) and 65 asymptomatic carriage (blue) *S. epidermidis* isolates not used for GWAS or phenotyping. The x-axis indicate individual isolates, ordered by their overall amount of associated and correlated kmers detected. The amount of kmers found in each isolate is indicated in the y-axes. (B) Distribution and frequency plot of all k-mer prevalence values for infection (red) and asymptomatic carriage isolates (blue). Error bars indicate s.d. Numbers highlight three different prevalence groups of isolates on both panels.

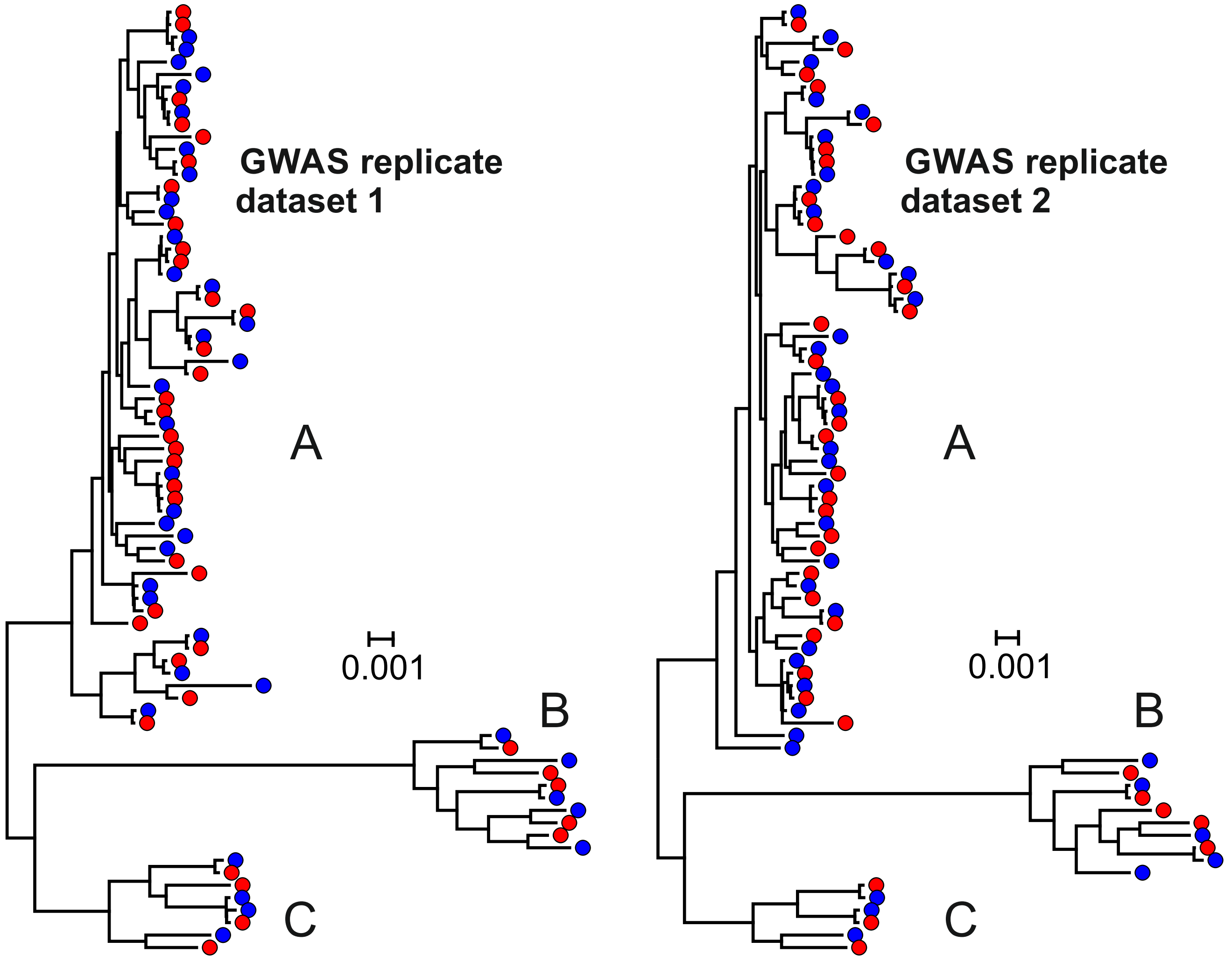

Supplementary Figure 6. Phylogenies reconstructed using ClonalFrameML and used as guiding trees for two GWAS technical replicate runs. No isolate is present in the two phylogenies. The letters indicate broad lineage designations.

Supplementary References.

1. Meric G, *et al.* (2014) A reference pan-genome approach to comparative bacterial genomics: identification of novel epidemiological markers in pathogenic Campylobacter. *PLoS One* 9(3):e92798.
2. Aziz RK, *et al.* (2008) The RAST Server: rapid annotations using subsystems technology. *Bmc Genomics* 9:75.
3. Overbeek R, *et al.* (2014) The SEED and the Rapid Annotation of microbial genomes using Subsystems Technology (RAST). *Nucleic Acids Res* 42(1):D206-214.
4. Pascoe B, *et al.* (2015) Enhanced biofilm formation and multi-host transmission evolve from divergent genetic backgrounds in Campylobacter jejuni. *Environ Microbiol*.
5. Sheppard SK, *et al.* (2013) Genome-wide association study identifies vitamin B5 biosynthesis as a host specificity factor in Campylobacter. *PNAS* 110(29):11923-11927.
6. Meric G, Kemsley EK, Falush D, Saggers EJ, & Lucchini S (2013) Phylogenetic distribution of traits associated with plant colonization in Escherichia coli. *Environmental microbiology* 15(2):487-501.
7. Pascoe B, *et al.* (2015) Enhanced biofilm formation and multi-host transmission evolve from divergent genetic backgrounds in Campylobacter jejuni. *Environ Microbiol* 17(11):4779-4789.
8. Laabei M, Jamieson WD, Yang Y, van den Elsen J, & Jenkins AT (2014) Investigating the lytic activity and structural properties of Staphylococcus aureus phenol soluble modulin (PSM) peptide toxins. *Biochim Biophys Acta* 1838(12):3153-3161.
9. Laabei M, Jamieson WD, Massey RC, & Jenkins AT (2014) Staphylococcus aureus interaction with phospholipid vesicles--a new method to accurately determine accessory gene regulator (agr) activity. *PLoS One* 9(1):e87270.
10. Laabei M (2014) Using phospholipid vesicles to assay bacterial lytic agents, examining factors and identifying virulence loci which alter toxin production in Staphylococcus aureus. (University of Bath).
11. Miragaia M, Thomas JC, Couto I, Enright MC, & de Lencastre H (2007) Inferring a population structure for Staphylococcus epidermidis from multilocus sequence typing data. *J Bacteriol* 189(6):2540-2552.
12. Boukamp P, *et al.* (1988) Normal keratinization in a spontaneously immortalized aneuploid human keratinocyte cell line. *J Cell Biol* 106(3):761-771.
13. Buttner H, Mack D, & Rohde H (2015) Structural basis of Staphylococcus epidermidis biofilm formation: mechanisms and molecular interactions. *Frontiers in cellular and infection microbiology* 5:14.
14. Mack D (1999) Molecular mechanisms of Staphylococcus epidermidis biofilm formation. *J Hosp Infect* 43 Suppl:S113-125.
15. Miragaia M, Couto I, & de Lencastre H (2005) Genetic diversity among methicillin-resistant Staphylococcus epidermidis (MRSE). *Microb Drug Resist* 11(2):83-93.
16. Collins J, Buckling A, & Massey RC (2008) Identification of factors contributing to T-cell toxicity of Staphylococcus aureus clinical isolates. *J Clin Microbiol* 46(6):2112-2114.
17. Betjes MG, *et al.* (1993) Interleukin-8 production by human peritoneal mesothelial cells in response to tumor necrosis factor-alpha, interleukin-1, and medium conditioned by macrophages cocultured with Staphylococcus epidermidis. *J Infect Dis* 168(5):1202-1210.
18. Stevens NT, *et al.* (2009) Staphylococcus epidermidis polysaccharide intercellular adhesin induces IL-8 expression in human astrocytes via a mechanism involving TLR2. *Cell Microbiol* 11(3):421-432.

19. Sachse F, von Eiff C, Becker K, Steinhoff M, & Rudack C (2008) Proinflammatory impact of Staphylococcus epidermidis on the nasal epithelium quantified by IL-8 and GRO-alpha responses in primary human nasal epithelial cells. *Int Arch Allergy Immunol* 145(1):24-32.
20. Yang HC, *et al.* (2015) Glucose 6-phosphate dehydrogenase knockdown enhances IL-8 expression in HepG2 cells via oxidative stress and NF-kappaB signaling pathway. *J Inflamm (Lond)* 12:34.
21. Hsieh YT, *et al.* (2013) Glucose-6-phosphate dehydrogenase (G6PD)-deficient epithelial cells are less tolerant to infection by Staphylococcus aureus. *PLoS One* 8(11):e79566.
22. Sinha B, *et al.* (2000) Heterologously expressed Staphylococcus aureus fibronectin-binding proteins are sufficient for invasion of host cells. *Infect Immun* 68(12):6871-6878.
23. Heyer G, *et al.* (2002) Staphylococcus aureus agr and sarA functions are required for invasive infection but not inflammatory responses in the lung. *Infect Immun* 70(1):127-133.
24. Jung K, Luthje P, Lundahl J, & Brauner A (2011) Low immunogenicity allows Staphylococcus epidermidis to cause PD peritonitis. *Perit Dial Int* 31(6):672-678.
25. Fluckiger U, *et al.* (2005) Biofilm formation, icaADBC transcription, and polysaccharide intercellular adhesin synthesis by staphylococci in a device-related infection model. *Infect Immun* 73(3):1811-1819.
26. Schaeffer CR, *et al.* (2015) Accumulation-Associated Protein Enhances Staphylococcus epidermidis Biofilm Formation under Dynamic Conditions and Is Required for Infection in a Rat Catheter Model. *Infect Immun* 83(1):214-226.
27. Sadykov MR, *et al.* (2008) Tricarboxylic acid cycle-dependent regulation of Staphylococcus epidermidis polysaccharide intercellular adhesin synthesis. *J Bacteriol* 190(23):7621-7632.
28. Scovill WH, Schreier HJ, & Bayles KW (1996) Identification and characterization of the pckA gene from Staphylococcus aureus. *J Bacteriol* 178(11):3362-3364.
29. Qin Z, *et al.* (2007) Antimicrobial activities of YycG histidine kinase inhibitors against Staphylococcus epidermidis biofilms. *FEMS Microbiol Lett* 273(2):149-156.
30. Hu C, Xiong N, Zhang Y, Rayner S, & Chen S (2012) Functional characterization of lipase in the pathogenesis of Staphylococcus aureus. *Biochem Biophys Res Commun* 419(4):617-620.
31. Diep BA, *et al.* (2006) Complete genome sequence of USA300, an epidemic clone of community-acquired meticillin-resistant Staphylococcus aureus. *Lancet* 367(9512):731-739.
32. Miragaia M, *et al.* (2009) Genetic diversity of arginine catabolic mobile element in Staphylococcus epidermidis. *PLoS One* 4(11):e7722.
33. Diep BA, *et al.* (2008) The arginine catabolic mobile element and staphylococcal chromosomal cassette mec linkage: convergence of virulence and resistance in the USA300 clone of methicillin-resistant Staphylococcus aureus. *J Infect Dis* 197(11):1523-1530.
34. Goering RV, *et al.* (2007) Epidemiologic distribution of the arginine catabolic mobile element among selected methicillin-resistant and methicillin-susceptible Staphylococcus aureus isolates. *J Clin Microbiol* 45(6):1981-1984.
35. Watkins RR, David MZ, & Salata RA (2012) Current concepts on the virulence mechanisms of meticillin-resistant Staphylococcus aureus. *J Med Microbiol* 61(Pt 9):1179-1193.

36. Laabei M, *et al.* (2014) Predicting the virulence of MRSA from its genome sequence. *Genome research* 24(5):839-849.
37. Jenkins R, Burton N, & Cooper R (2011) Effect of manuka honey on the expression of universal stress protein A in meticillin-resistant Staphylococcus aureus. *Int J Antimicrob Agents* 37(4):373-376.
38. Zhang YQ, *et al.* (2003) Genome-based analysis of virulence genes in a non-biofilm-forming Staphylococcus epidermidis strain (ATCC 12228). *Mol Microbiol* 49(6):1577-1593.
39. Hanssen AM & Ericson Sollid JU (2006) SCCmec in staphylococci: genes on the move. *FEMS Immunol Med Microbiol* 46(1):8-20.
