# Supplementary material for: Disease-associated genotypes of the commensal skin bacterium Staphylococcus epidermidis
Source: Nat Commun. 2018 Nov 28;9:5034. doi: 10.1038/s41467-018-07368-7 (PMC6261936; doi:10.1038/s41467-018-07368-7)
Supplement: Supplementary file 3 — Description of Additional Supplementary Files [file 41467_2018_7368_MOESM3_ESM.pdf]

Description of Additional Supplementary Files

File Name: Supplementary Data 1

Description: List of 61 genes containing pathogenicity-associated 30-mers correlated with one of 4 in vitro secondary phenotype scores

File Name: Supplementary Data 2

Description: Isolates and genomes used in this study

File Name: Supplementary Data 3

Description: List of 636 genes associated with infection in S. epidermidis

File Name: Supplementary Data 4

Description: COG functional annotation of the reference genome ATCC12228, the pangenome list of 415 S. epidermidis strains, the GWAS hits and the genes statistically correlated with in vitro phenotypes

File Name: Supplementary Data 5

Description: Assembly metrics for isolates sequenced as part of this study

File Name: Supplementary Data 6

Description: Summary of in vitro phenotype scores used for functional filtering of GWAS results

File Name: Supplementary Data 7

Description: List of genomes available on NCBI including those used for validation of risk prediction analyses
