# Supplementary material for: Disease-associated genotypes of the commensal skin bacterium Staphylococcus epidermidis
Source: Nat Commun. 2018 Nov 28;9:5034. doi: 10.1038/s41467-018-07368-7 (PMC6261936; doi:10.1038/s41467-018-07368-7)
Supplement: Supplementary file 11 — Reporting Summary [file 41467_2018_7368_MOESM11_ESM.pdf]

nature research

Corresponding author(s): NCOMMS-18-09419A

Reporting Summary

Nature Research wishes to improve the reproducibility of the work that we publish. This form provides structure for consistency and transparency in reporting. For further information on Nature Research policies, see Authors & Referees and the Editorial Policy Checklist.

Statistical parameters

When statistical analyses are reported, confirm that the following items are present in the relevant location (e.g. figure legend, table legend, main text, or Methods section).

n/a	Confirmed	
☐	☒	The exact sample size (*n*) for each experimental group/condition, given as a discrete number and unit of measurement
☐	☒	An indication of whether measurements were taken from distinct samples or whether the same sample was measured repeatedly
☐	☒	The statistical test(s) used AND whether they are one- or two-sided<br>*Only common tests should be described solely by name; describe more complex techniques in the Methods section.*
☐	☒	A description of all covariates tested
☒	☐	A description of any assumptions or corrections, such as tests of normality and adjustment for multiple comparisons
☐	☒	A full description of the statistics including central tendency (e.g. means) or other basic estimates (e.g. regression coefficient) AND variation (e.g. standard deviation) or associated estimates of uncertainty (e.g. confidence intervals)
☐	☒	For null hypothesis testing, the test statistic (e.g. *F*, *t*, *r*) with confidence intervals, effect sizes, degrees of freedom and *P* value noted<br>*Give P values as exact values whenever suitable.*
☒	☐	For Bayesian analysis, information on the choice of priors and Markov chain Monte Carlo settings
☒	☐	For hierarchical and complex designs, identification of the appropriate level for tests and full reporting of outcomes
☒	☐	Estimates of effect sizes (e.g. Cohen's *d*, Pearson's *r*), indicating how they were calculated
☐	☒	Clearly defined error bars<br>*State explicitly what error bars represent (e.g. SD, SE, CI)*

Our web collection on statistics for biologists may be useful.

Software and code

Policy information about availability of computer code

Data collection	Isolate genome and metadata was archived in BIGdb. The BIGSdb software is written in Perl, also utilizing some client-side Javascript. It runs on Linux using the Apache web server and PostgreSQL database. BIGSdb is open-source software, published under the GNU General Public Licence version 3.
Data analysis	A list of all commercial, custom and open source code used for analysis in this study is given below. Reference numbers are those given in the manuscript reference list.<br>1. A S. epidermidis coding sequence pangenome gene list was constructed by automatic annotation of all genomes from the dataset using the RAST/SEED system (39) and the WebMGA COG annotation server (40).<br>2. Core gene sequences were individually aligned, using MUSCLE (46). Trees were reconstructed using an approximation of maximum-likelihood phylogenetics in FastTree2 (47). This tree was used as an input for ClonalFrameML (48).<br>3. The GWAS pipeline used custom scripts available on https://github.com/sheppardlab/pGWAS.<br>4. Consistency index was calculated using the R Phangorn package (64).<br>5. The random forest analyses were done using the TreeBagger function in the Statistics and Machine Learning Toolbox in MATLAB R2016b.

For manuscripts utilizing custom algorithms or software that are central to the research but not yet described in published literature, software must be made available to editors/reviewers upon request. We strongly encourage code deposition in a community repository (e.g. GitHub). See the Nature Research guidelines for submitting code & software for further information.

Data

Policy information about availability of data

All manuscripts must include a data availability statement. This statement should provide the following information, where applicable:

- Accession codes, unique identifiers, or web links for publicly available datasets
- A list of figures that have associated raw data
- A description of any restrictions on data availability

All scripts and example input and output files are available on: https://github.com/sheppardlab/pGWAS and Figshare (doi:10.6084/m9.figshare.5856861). Short read sequence data for all 241 isolates sequenced in this study are deposited in the SRA and can be found associated with BioProject: PRNJA433155 (https://www.ncbi.nlm.nih.gov/). Assembled genomes are also available on figshare (doi: 10.6084/m9.figshare.7058543) or through our publicly available BIGSdb: https://sheppardlab.com/resources/. NCBI genome accession numbers for isolates in the validation dataset are included in Table S6.

Field-specific reporting

Please select the best fit for your research. If you are not sure, read the appropriate sections before making your selection.

☒ Life sciences ☐ Behavioural & social sciences ☐ Ecological, evolutionary & environmental sciences

For a reference copy of the document with all sections, see nature.com/authors/policies/ReportingSummary-flat.pdf

Life sciences study design

All studies must disclose on these points even when the disclosure is negative.

Sample size	Samples were collected as part of UKRI funded programs. No formal power calculation was carried out as the population genomic structure of S. epidermidis was largely unknown before this study. All invasive samples that were collected during the study period were sequenced. Carriage samples were collected and as many were sequenced as possible given the funding limitations inherent in the study.
Data exclusions	Isolates for which a genome sequencing failed were not included.
Replication	All attempts at replication were successful.
Randomization	Throughout the study, isolate organization into groups was principally carried out based upon metadata. Specifically isolation source: 'invasive' strains from infected indwelling devices and blood, and 'carriage' strains from the skin and nasal pharynx of healthy volunteers.
Blinding	This is relevant to association studies but it is not possible in GWAS experiments because phenotype (source) data is necessarily included a priori in order to detect elements that are over represented in one group or another (compared to expected frequencies based upon the tree).

Reporting for specific materials, systems and methods

Materials & experimental systems

n/a	Involved in the study	
☒	☐	Unique biological materials
☒	☐	Antibodies
☐	☒	Eukaryotic cell lines
☒	☐	Palaeontology
☒	☐	Animals and other organisms
☐	☒	Human research participants

Methods

n/a	Involved in the study	
☒	☐	ChIP-seq
☒	☐	Flow cytometry
☒	☐	MRI-based neuroimaging

Eukaryotic cell lines

Policy information about cell lines

Cell line source(s)	HaCaT keratinocytes were from immortalized human skin epithelial cell lines obtained from ATCC.
Authentication	Cell lines were not specifically authenticated for this study, apart from the ATCC assurances.

Mycoplasma contamination	Cell lines were cultured in control experiments to check for contamination. No contamination was recorded.
Commonly misidentified lines (See ICLAC register)	No commonly misidentified cell lines were used in this study.

Human research participants

Policy information about studies involving human research participants

Population characteristics	Asymptomatic carriage isolates were sampled from healthy volunteers in Swansea University (UK) in 2012. Swabs were collected from, and samples were deposited in, a container in the laboratory. Body site and date were written on the tube. This population included male and female staff and students. Volunteers remained anonymous throughout and no metadata was collected from participants.
Recruitment	Participants were recruited through word of mouth and by posting notices asking for volunteers. This was carried out at a university (Swansea) so there may be a bias towards people of student age although researchers, lecturers and other staff may have participated. I confirm that we complied with all relevant ethical regulations. Volunteers gave informed consent, as assessed by the local Human Tissue Act committee (Wales REC 6) at the Swansea University Medical School (ref: #13/WA/0190).
